# (1*H*-Pyrrol-2-ylmethylidene)(3-{[(1*H*-pyrrol-2-ylmethylidene)amino]methyl}benzyl)amine

**DOI:** 10.1107/S1600536810046799

**Published:** 2010-11-17

**Authors:** Dong Wan Kim, Tae Ho Kim, Jineun Kim, Jae Sang Kim

**Affiliations:** a Department of Chemistry and Research Institute of Natural Sciences, Gyeongsang, National University, Jinju 660-701, Republic of Korea

## Abstract

In the title compound, C_18_H_18_N_4_, the dihedral angles between the pyrrole rings and the phenyl ring are 85.07 (8)° and 77.13 (9)°. Inter­molecular N—H⋯N hydrogen bonds contribute to the stabilization of the crystal packing.

## Related literature

For the synthesis of the title compound, see: Chakravorty & Holm (1964 ▶); Jasat & Dolphin, (1997 ▶). For related structures, see: Nativi *et al.* (2007 ▶).
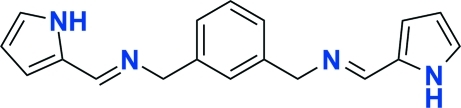


## Experimental

### 

#### Crystal data

C_18_H_18_N_4_
*M* *_r_* = 290.36
Monoclinic, 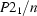

*a* = 5.0010 (6) Å
*b* = 17.271 (2) Å
*c* = 17.764 (2) Å
β = 96.128 (9)°
*V* = 1525.5 (3) Å^3^
*Z* = 4
Mo *K*α radiation
μ = 0.08 mm^−1^
*T* = 173 K
0.15 × 0.05 × 0.02 mm

#### Data collection

Bruker APEXII CCD diffractometer
Absorption correction: multi-scan (*SADABS*; Sheldrick, 1996 ▶) *T*_min_ = 0.988, *T*_max_ = 0.998
12454 measured reflections
2990 independent reflections
1550 reflections with *I* > 2σ(*I*)
*R*_int_ = 0.087

#### Refinement

*R*[*F* ^2^ > 2σ(*F* ^2^)] = 0.060
*wR*(*F* ^2^) = 0.141
*S* = 0.98
2990 reflections
200 parameters
H-atom parameters constrained
Δρ_max_ = 0.20 e Å^−3^
Δρ_min_ = −0.18 e Å^−3^

### 

Data collection: *APEX2* (Bruker, 2006 ▶); cell refinement: *SAINT* (Bruker, 2006 ▶); data reduction: *SAINT*; program(s) used to solve structure: *SHELXTL* (Sheldrick, 2008 ▶); program(s) used to refine structure: *SHELXTL*; molecular graphics: *SHELXTL* and *DIAMOND* (Brandenburg, 1998 ▶); software used to prepare material for publication: *SHELXTL*.

## Supplementary Material

Crystal structure: contains datablocks global, I. DOI: 10.1107/S1600536810046799/jh2230sup1.cif

Structure factors: contains datablocks I. DOI: 10.1107/S1600536810046799/jh2230Isup2.hkl

Additional supplementary materials:  crystallographic information; 3D view; checkCIF report

## Figures and Tables

**Table 1:** Hydrogen-bond geometry (Å, °)

*D*—H⋯*A*	*D*—H	H⋯*A*	*D*⋯*A*	*D*—H⋯*A*
N1—H1⋯N3^i^	0.88	2.17	2.993 (3)	156
N4—H4⋯N2^ii^	0.88	2.12	2.949 (3)	158

Symmetry codes: (i) 
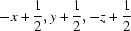
; (ii) 
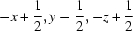
.

**Fig. 1.**
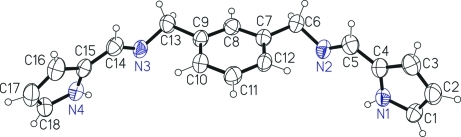
The molecular structure of the title compound, showing displacement ellipsoids drawn at the 50% probability level.

**Fig. 2.**
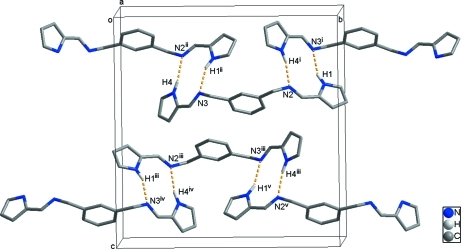
Crystal packing of the title compound with intermolecular N—H···N hydrogen bonds shown as dashed lines. H atoms not involved in intermolecular interactions have been omitted for clarity. [Symmetry codes: (i) -x + 1/2, y + 1/2, -z + 1/2; (ii) -x + 1/2, y - 1/2, -z + 1/2; (iii) -x + 1, -y + 1, -z + 1; (iv) x + 1/2, -y + 1/2, z + 1/2; (v) x + 1/2, -y - 1.5, z + 1/2.]

## supplementary crystallographic information

### Comment

The design and synthesis of supramolcular ligands are based on the ability to organize the binding site and size complementarities in a proper way. Especially, metal ions assisted self-assembly are one of most powerful approaches to supramolecular architectures. For examples, the N4 type of tetradentate ligands, pyrrole-2-yl Schiff base and pyrrole-2-ylmethylene amines have been known for a long time (Chakravorty & Holm, 1964; Jasat & Dolphin, 1997).

In the asymmetric unit (Fig. 1), the dihedral angle between the pyrrole ring plane system and phenyl ring plane are 85.07 (8)° and 77.13 (9)°. All bond lengths and bond angles of pyrrole-2-ylmethylene group are are comparable to those observed in similar structures (Nativi *et al.*, 2007).

In the crystal structure, intermolecular N—H···N hydrogen bonds are observed. These interactinos contribute to stabilization of the packing (Fig. 2).

### Experimental

Pyrrole-2-carbaldehyde (1.9 g, 20 mmol) and 1,3-phenylenedimethanamine (1.36 g, 10 mmol) were dissolved in ethanol (20 ml). The mixture strirred for a while, and then a few drops of acetic acid was added. After about 30 min, a light yellow precipitate was observed. After about 20 min, the precipitate obtained from filtration was washed with ethanol, dried in vacuum. Slow evaporation of a solution in CH_2_Cl_2_ gave single crystals suitable for X-ray analysis.

FT—IR (KBr disk) 3166, 3110, 2830, 1641 cm^-1. 1^H NMR (300 MHz, DMSO-d_6_) 4.7 (s, 2H), 6.1 (d, 2H, J=4.2 Hz), 6.5 (t, 2H, J=2.8 Hz), 6.9 (d, 2H, J=4.2 Hz), 7.3 (m, 4H), 8.2 (s, 2H), 11.4 (broad s, 2H). ^13^C NMR (75 MHz, DMSO-d_6_) 39.1, 64.3, 109.3, 114.2, 122.6, 126.9, 128.1, 130.4, 140.6, 152.9. EI—MS (m/*z*): 290 (*M*^+^).

### Refinement

All H-atoms were positioned geometrically and refined using a riding model with d(N—H) = 0.88 Å, *U*_iso_(H) = 1.2*U*_eq_(C) for pyrrole N, d(C—H) = 0.95 Å, *U*_iso_(H) = 1.2*U*_eq_(C) for aromatic C and d(C—H) = 0.99 Å, *U*_iso_(H) = 1.2*U*_eq_(C) for CH_2_ groups.

### Crystal data

**Table:**

C_18_H_18_N_4_	*F*(000) = 616
*M**_r_* = 290.36	*D*_x_ = 1.264 Mg m^−^^3^
Monoclinic, *P*2_1_/*n*	Mo *K*α radiation, λ = 0.71073 Å
Hall symbol: -P 2yn	Cell parameters from 867 reflections
*a* = 5.0010 (6) Å	θ = 2.3–18.8°
*b* = 17.271 (2) Å	µ = 0.08 mm^−^^1^
*c* = 17.764 (2) Å	*T* = 173 K
β = 96.128 (9)°	Plate, yellow
*V* = 1525.5 (3) Å^3^	0.15 × 0.05 × 0.02 mm
*Z* = 4	

### Data collection

**Table:**

Bruker APEXII CCD diffractometer	2990 independent reflections
Radiation source: fine-focus sealed tube	1550 reflections with *I* > 2σ(*I*)
graphite	*R*_int_ = 0.087
φ and ω scans	θ_max_ = 26.0°, θ_min_ = 1.7°
Absorption correction: multi-scan (*SADABS*; Sheldrick, 1996)	*h* = −6→6
*T*_min_ = 0.988, *T*_max_ = 0.998	*k* = −21→19
12454 measured reflections	*l* = −21→21

### Refinement

**Table:**

Refinement on *F*^2^	Secondary atom site location: difference Fourier map
Least-squares matrix: full	Hydrogen site location: inferred from neighbouring sites
*R*[*F*^2^ > 2σ(*F*^2^)] = 0.060	H-atom parameters constrained
*wR*(*F*^2^) = 0.141	*w* = 1/[σ^2^(*F*_o_^2^) + (0.0531*P*)^2^] where *P* = (*F*_o_^2^ + 2*F*_c_^2^)/3
*S* = 0.98	(Δ/σ)_max_ < 0.001
2990 reflections	Δρ_max_ = 0.20 e Å^−^^3^
200 parameters	Δρ_min_ = −0.17 e Å^−^^3^
0 restraints	Extinction correction: *SHELXL*, Fc^*^=kFc[1+0.001xFc^2^λ^3^/sin(2θ)]^-1/4^
Primary atom site location: structure-invariant direct methods	Extinction coefficient: 0.0061 (15)

### Special details

**Table:**

Geometry. All e.s.d.'s (except the e.s.d. in the dihedral angle between two l.s. planes) are estimated using the full covariance matrix. The cell e.s.d.'s are taken into account individually in the estimation of e.s.d.'s in distances, angles and torsion angles; correlations between e.s.d.'s in cell parameters are only used when they are defined by crystal symmetry. An approximate (isotropic) treatment of cell e.s.d.'s is used for estimating e.s.d.'s involving l.s. planes.
Refinement. Refinement of *F*^2^ against ALL reflections. The weighted *R*-factor *wR* and goodness of fit *S* are based on *F*^2^, conventional *R*-factors *R* are based on *F*, with *F* set to zero for negative *F*^2^. The threshold expression of *F*^2^ > σ(*F*^2^) is used only for calculating *R*-factors(gt) *etc*. and is not relevant to the choice of reflections for refinement. *R*-factors based on *F*^2^ are statistically about twice as large as those based on *F*, and *R*- factors based on ALL data will be even larger.

### Fractional atomic coordinates and isotropic or equivalent isotropic displacement parameters (Å^2^)

**Table:**

	*x*	*y*	*z*	*U*_iso_*/*U*_eq_	
N1	0.1887 (4)	0.93308 (13)	0.30396 (12)	0.0471 (6)	
H1	0.2430	0.9034	0.2683	0.057*	
N2	−0.1228 (4)	0.78669 (13)	0.30188 (12)	0.0479 (6)	
N3	0.0866 (4)	0.37738 (12)	0.33592 (12)	0.0443 (6)	
N4	0.4888 (4)	0.24887 (12)	0.35156 (12)	0.0466 (6)	
H4	0.4867	0.2670	0.3052	0.056*	
C1	0.2853 (6)	1.00448 (17)	0.32310 (17)	0.0555 (8)	
H1A	0.4232	1.0307	0.3004	0.067*	
C2	0.1523 (6)	1.03243 (18)	0.38019 (17)	0.0597 (8)	
H2	0.1807	1.0812	0.4045	0.072*	
C3	−0.0330 (6)	0.97645 (17)	0.39655 (15)	0.0539 (8)	
H3	−0.1554	0.9803	0.4338	0.065*	
C4	−0.0070 (5)	0.91461 (16)	0.34911 (14)	0.0454 (7)	
C5	−0.1556 (6)	0.84397 (16)	0.34583 (15)	0.0488 (7)	
H5	−0.2909	0.8389	0.3792	0.059*	
C6	−0.2874 (5)	0.71839 (15)	0.31250 (16)	0.0483 (7)	
H6A	−0.3806	0.7024	0.2630	0.058*	
H6B	−0.4261	0.7318	0.3461	0.058*	
C7	−0.1203 (5)	0.65149 (15)	0.34658 (14)	0.0399 (7)	
C8	−0.1874 (5)	0.57583 (16)	0.32711 (14)	0.0436 (7)	
H8	−0.3368	0.5664	0.2906	0.052*	
C9	−0.0432 (5)	0.51348 (15)	0.35929 (14)	0.0406 (7)	
C10	0.1738 (6)	0.52757 (16)	0.41134 (14)	0.0461 (7)	
H10	0.2763	0.4856	0.4336	0.055*	
C11	0.2443 (5)	0.60305 (16)	0.43154 (14)	0.0476 (7)	
H11	0.3950	0.6126	0.4676	0.057*	
C12	0.0964 (6)	0.66413 (16)	0.39942 (15)	0.0450 (7)	
H12	0.1448	0.7156	0.4140	0.054*	
C13	−0.1352 (5)	0.43160 (15)	0.33830 (17)	0.0519 (8)	
H13A	−0.2540	0.4130	0.3756	0.062*	
H13B	−0.2420	0.4326	0.2881	0.062*	
C14	0.1251 (6)	0.33165 (16)	0.39264 (15)	0.0469 (7)	
H14	0.0085	0.3373	0.4311	0.056*	
C15	0.3263 (5)	0.27306 (15)	0.40368 (14)	0.0425 (7)	
C16	0.3911 (6)	0.23031 (17)	0.46802 (16)	0.0556 (8)	
H16	0.3085	0.2341	0.5136	0.067*	
C17	0.5993 (6)	0.18029 (17)	0.45480 (17)	0.0592 (8)	
H17	0.6864	0.1444	0.4898	0.071*	
C18	0.6545 (6)	0.19249 (16)	0.38240 (18)	0.0552 (8)	
H18	0.7867	0.1660	0.3576	0.066*	

### Atomic displacement parameters (Å^2^)

**Table:**

	*U*^11^	*U*^22^	*U*^33^	*U*^12^	*U*^13^	*U*^23^
N1	0.0528 (14)	0.0354 (14)	0.0530 (14)	0.0013 (12)	0.0052 (12)	−0.0062 (11)
N2	0.0588 (15)	0.0349 (14)	0.0505 (14)	−0.0004 (12)	0.0073 (12)	0.0055 (11)
N3	0.0514 (14)	0.0296 (13)	0.0513 (14)	−0.0039 (11)	0.0031 (11)	−0.0047 (11)
N4	0.0575 (14)	0.0362 (14)	0.0463 (13)	−0.0017 (12)	0.0065 (11)	0.0033 (10)
C1	0.0540 (18)	0.0411 (19)	0.070 (2)	−0.0056 (15)	−0.0008 (16)	−0.0095 (15)
C2	0.062 (2)	0.0461 (19)	0.068 (2)	0.0031 (17)	−0.0030 (17)	−0.0154 (16)
C3	0.0631 (19)	0.054 (2)	0.0443 (17)	0.0067 (17)	0.0046 (14)	−0.0067 (14)
C4	0.0553 (18)	0.0410 (18)	0.0398 (15)	0.0019 (15)	0.0043 (14)	0.0011 (12)
C5	0.0599 (19)	0.0463 (19)	0.0410 (16)	0.0061 (15)	0.0088 (14)	0.0070 (13)
C6	0.0526 (17)	0.0369 (17)	0.0555 (18)	−0.0025 (14)	0.0066 (14)	0.0048 (13)
C7	0.0473 (17)	0.0364 (17)	0.0369 (15)	−0.0013 (13)	0.0083 (14)	−0.0002 (12)
C8	0.0428 (16)	0.0437 (19)	0.0439 (16)	−0.0025 (14)	0.0022 (13)	−0.0035 (13)
C9	0.0437 (16)	0.0348 (17)	0.0429 (15)	−0.0033 (14)	0.0031 (14)	−0.0037 (12)
C10	0.0557 (18)	0.0346 (17)	0.0477 (17)	−0.0007 (14)	0.0042 (15)	−0.0008 (12)
C11	0.0494 (17)	0.0438 (19)	0.0482 (17)	−0.0049 (15)	−0.0012 (14)	−0.0069 (13)
C12	0.0542 (19)	0.0311 (17)	0.0510 (17)	−0.0025 (14)	0.0125 (15)	−0.0026 (13)
C13	0.0505 (17)	0.0362 (18)	0.068 (2)	−0.0004 (15)	0.0022 (15)	−0.0109 (14)
C14	0.0583 (19)	0.0380 (17)	0.0457 (17)	−0.0077 (15)	0.0109 (14)	−0.0087 (13)
C15	0.0538 (17)	0.0310 (16)	0.0423 (16)	−0.0074 (14)	0.0032 (14)	−0.0031 (12)
C16	0.076 (2)	0.0437 (19)	0.0464 (18)	−0.0058 (17)	0.0027 (15)	−0.0001 (14)
C17	0.074 (2)	0.0400 (19)	0.060 (2)	−0.0024 (17)	−0.0114 (17)	0.0083 (15)
C18	0.0548 (19)	0.0394 (19)	0.070 (2)	0.0037 (15)	0.0022 (16)	0.0057 (15)

### Geometric parameters (Å, °)

**Table:**

N1—C1	1.354 (3)	C7—C12	1.373 (4)
N1—C4	1.368 (3)	C7—C8	1.384 (3)
N1—H1	0.8800	C8—C9	1.385 (3)
N2—C5	1.282 (3)	C8—H8	0.9500
N2—C6	1.462 (3)	C9—C10	1.370 (4)
N3—C14	1.279 (3)	C9—C13	1.521 (3)
N3—C13	1.456 (3)	C10—C11	1.388 (4)
N4—C18	1.356 (3)	C10—H10	0.9500
N4—C15	1.361 (3)	C11—C12	1.377 (3)
N4—H4	0.8800	C11—H11	0.9500
C1—C2	1.359 (4)	C12—H12	0.9500
C1—H1A	0.9500	C13—H13A	0.9900
C2—C3	1.391 (4)	C13—H13B	0.9900
C2—H2	0.9500	C14—C15	1.426 (4)
C3—C4	1.375 (4)	C14—H14	0.9500
C3—H3	0.9500	C15—C16	1.370 (4)
C4—C5	1.426 (4)	C16—C17	1.392 (4)
C5—H5	0.9500	C16—H16	0.9500
C6—C7	1.514 (4)	C17—C18	1.360 (4)
C6—H6A	0.9900	C17—H17	0.9500
C6—H6B	0.9900	C18—H18	0.9500
			
C1—N1—C4	108.9 (2)	C9—C8—H8	119.0
C1—N1—H1	125.6	C10—C9—C8	118.7 (2)
C4—N1—H1	125.6	C10—C9—C13	121.8 (2)
C5—N2—C6	115.7 (2)	C8—C9—C13	119.4 (2)
C14—N3—C13	115.2 (2)	C9—C10—C11	120.2 (3)
C18—N4—C15	109.2 (2)	C9—C10—H10	119.9
C18—N4—H4	125.4	C11—C10—H10	119.9
C15—N4—H4	125.4	C12—C11—C10	120.1 (3)
N1—C1—C2	108.7 (3)	C12—C11—H11	119.9
N1—C1—H1A	125.6	C10—C11—H11	119.9
C2—C1—H1A	125.6	C7—C12—C11	120.8 (3)
C1—C2—C3	107.4 (3)	C7—C12—H12	119.6
C1—C2—H2	126.3	C11—C12—H12	119.6
C3—C2—H2	126.3	N3—C13—C9	113.2 (2)
C4—C3—C2	107.6 (3)	N3—C13—H13A	108.9
C4—C3—H3	126.2	C9—C13—H13A	108.9
C2—C3—H3	126.2	N3—C13—H13B	108.9
N1—C4—C3	107.4 (2)	C9—C13—H13B	108.9
N1—C4—C5	125.3 (2)	H13A—C13—H13B	107.8
C3—C4—C5	127.3 (3)	N3—C14—C15	126.3 (2)
N2—C5—C4	125.8 (3)	N3—C14—H14	116.9
N2—C5—H5	117.1	C15—C14—H14	116.9
C4—C5—H5	117.1	N4—C15—C16	107.3 (3)
N2—C6—C7	111.9 (2)	N4—C15—C14	126.0 (2)
N2—C6—H6A	109.2	C16—C15—C14	126.7 (3)
C7—C6—H6A	109.2	C15—C16—C17	107.9 (3)
N2—C6—H6B	109.2	C15—C16—H16	126.0
C7—C6—H6B	109.2	C17—C16—H16	126.0
H6A—C6—H6B	107.9	C18—C17—C16	107.1 (3)
C12—C7—C8	118.3 (2)	C18—C17—H17	126.4
C12—C7—C6	120.9 (2)	C16—C17—H17	126.4
C8—C7—C6	120.8 (3)	N4—C18—C17	108.4 (3)
C7—C8—C9	122.0 (3)	N4—C18—H18	125.8
C7—C8—H8	119.0	C17—C18—H18	125.8
			
C4—N1—C1—C2	−0.2 (3)	C13—C9—C10—C11	−177.1 (2)
N1—C1—C2—C3	−0.2 (3)	C9—C10—C11—C12	0.0 (4)
C1—C2—C3—C4	0.6 (3)	C8—C7—C12—C11	0.6 (3)
C1—N1—C4—C3	0.6 (3)	C6—C7—C12—C11	178.6 (2)
C1—N1—C4—C5	−179.9 (3)	C10—C11—C12—C7	−0.7 (4)
C2—C3—C4—N1	−0.7 (3)	C14—N3—C13—C9	103.7 (3)
C2—C3—C4—C5	179.8 (3)	C10—C9—C13—N3	−36.8 (3)
C6—N2—C5—C4	176.1 (3)	C8—C9—C13—N3	145.5 (2)
N1—C4—C5—N2	2.1 (4)	C13—N3—C14—C15	179.4 (2)
C3—C4—C5—N2	−178.5 (3)	C18—N4—C15—C16	−0.5 (3)
C5—N2—C6—C7	−109.9 (3)	C18—N4—C15—C14	179.9 (3)
N2—C6—C7—C12	35.8 (3)	N3—C14—C15—N4	−9.2 (4)
N2—C6—C7—C8	−146.3 (2)	N3—C14—C15—C16	171.3 (3)
C12—C7—C8—C9	0.1 (3)	N4—C15—C16—C17	0.9 (3)
C6—C7—C8—C9	−177.9 (2)	C14—C15—C16—C17	−179.5 (3)
C7—C8—C9—C10	−0.7 (4)	C15—C16—C17—C18	−1.0 (3)
C7—C8—C9—C13	177.1 (2)	C15—N4—C18—C17	−0.1 (3)
C8—C9—C10—C11	0.6 (4)	C16—C17—C18—N4	0.7 (3)

### Hydrogen-bond geometry (Å, °)

**Table:**

*D*—H···*A*	*D*—H	H···*A*	*D*···*A*	*D*—H···*A*
N1—H1···N3^i^	0.88	2.17	2.993 (3)	156
N4—H4···N2^ii^	0.88	2.12	2.949 (3)	158

Symmetry codes: (i) −*x*+1/2, *y*+1/2, −*z*+1/2; (ii) −*x*+1/2, *y*−1/2, −*z*+1/2.
